# Left omental artery bleeding in two patients with segmental arterial mediolysis successfully isolated with coil embolization

**DOI:** 10.1186/s42155-020-00127-0

**Published:** 2020-07-19

**Authors:** Tomoya Nishiyama, Daisuke Yamada, Ken Oba, Yasuyuki Kurihara

**Affiliations:** 1 Department of Radiology, St. Luke’s International Hospital, 9-1, Akashi-chou, Chuo-ku, Tokyo, 104-8560 Japan; 2 Department of Radiology, Graduate School of Medicine, The University of Tokyo, 7-3-1, Hongo, Bunkyo-ku, Tokyo, 113-8655 Japan

**Keywords:** Segmental arterial mediolysis, Omental artery, Arterial collateral network, Isolation technique, Coil embolization

## Abstract

**Background:**

Segmental arterial mediolysis (SAM) is a rare, nonatherosclerotic, noninflammatory arteriopathy of unknown etiology, rarely involving omental artery (OA). No case reports have described left OA bleeding successfully treated with transarterial embolization (TAE) with coils. This report describes two cases of SAM-affected left OA bleeding successfully embolized using isolation technique with coils, recognizing the potential for the greater omentum to have arterial collateral network between OAs.

**Case presentation:**

Case 1.

A 55-year-old male with no significant past medical history presented with an acute abdomen. Contrast-enhanced computed tomography (CT) revealed possible hemorrhagic ascites involving the left portion of the greater omentum and dilated, stenotic change of the left OA with a possible hematoma. SAM-associated left OA bleeding was suspected. Given its acute-angled branching from a splenic artery or branch and long, tortuous catheter-trajectory, we used a triaxial catheter system. Left OA angiography revealed the proximal dilated, stenotic change and a distal pseudoaneurysm. Isolation was successfully performed with coils. Because he had no abdominal pain or progressive anemia, he was discharged on hospital day 5. Neither recurrence nor new SAM-associated findings were observed during two-years of follow-up.

Case 2.

A 60-year-old-man with no significant past medical history presented with an acute abdomen. CT revealed similar finding as Case 1. SAM-associated left OA bleeding was suspected. Left OA angiography revealed proximal dilated, stenotic change with distal occlusion. Despite having no signs of active bleeding, review of the CT and angiography findings suggested the left OA as the bleeding site. Given proximal embolization at this point could lead to incomplete hemostasis or rebleeding via the arterial collateral network between OAs, an attempt was made to navigate the microcatheter into the distal side beyond the occlusion. Distal left OA angiography confirmed that the distal OA over the occlusion was intact and directly communicated with a right OA arising from right gastroepiploic artery. The SAM-associated lesion was successfully isolated with coils. Because he had no abdominal pain or progressive anemia, he was transported to another hospital on hospital day 3. Neither recurrence nor new SAM-associated findings were observed during two-years follow-up.

**Conclusion:**

SAM can involve left OA and be controlled using an isolation technique with coils.

## Background

SAM is a rare, nonatherosclerotic, noninflammatory arteriopathy of unknown etiology and primarily involves visceral arteries, which can cause life-threatening bleeding (Slavin 2009; Kalva et al. 2011). SAM is vacuolization and lysis of the media that can lead to weakening of the media, formation of arterial wall gaps, and separation of the media from the adventitia; which can result in focal dissection, aneurysm formation, aneurysmal dilatation, stenosis, occlusion or bleeding (Slavin 2009; Kalva et al. 2011). Differential diagnosis includes many varieties of vasculitis including fibromuscular dysplasia (Slavin 2009; Kalva et al. 2011). There have been only three case reports describing SAM-affected OA bleeding (Heritz et al. 1990; Yasuoka et al. 2008; Rott and Boecker 2018). One case was treated with surgical intervention, while the other two failed TAE with coils (Yasuoka et al. 2008; Rott and Boecker 2018). For non-SAM OA bleeding associated with many underlying diseases and conditions, superselective cannulation into OA followed by TAE with coils has not been successfully performed (Tsuchiya et al. 2009; Matsumoto et al. 2011; Takahashi et al. 2012; Tajima et al. 2014; Nishiyama et al. 2018).

Herein, we describe two cases of left OA bleeding associated with SAM that were successfully embolized using an isolation technique with coils, taking into consideration the possible arterial collateral network between OAs.

## Case presentations

### Case 1

**Fig. 1:**
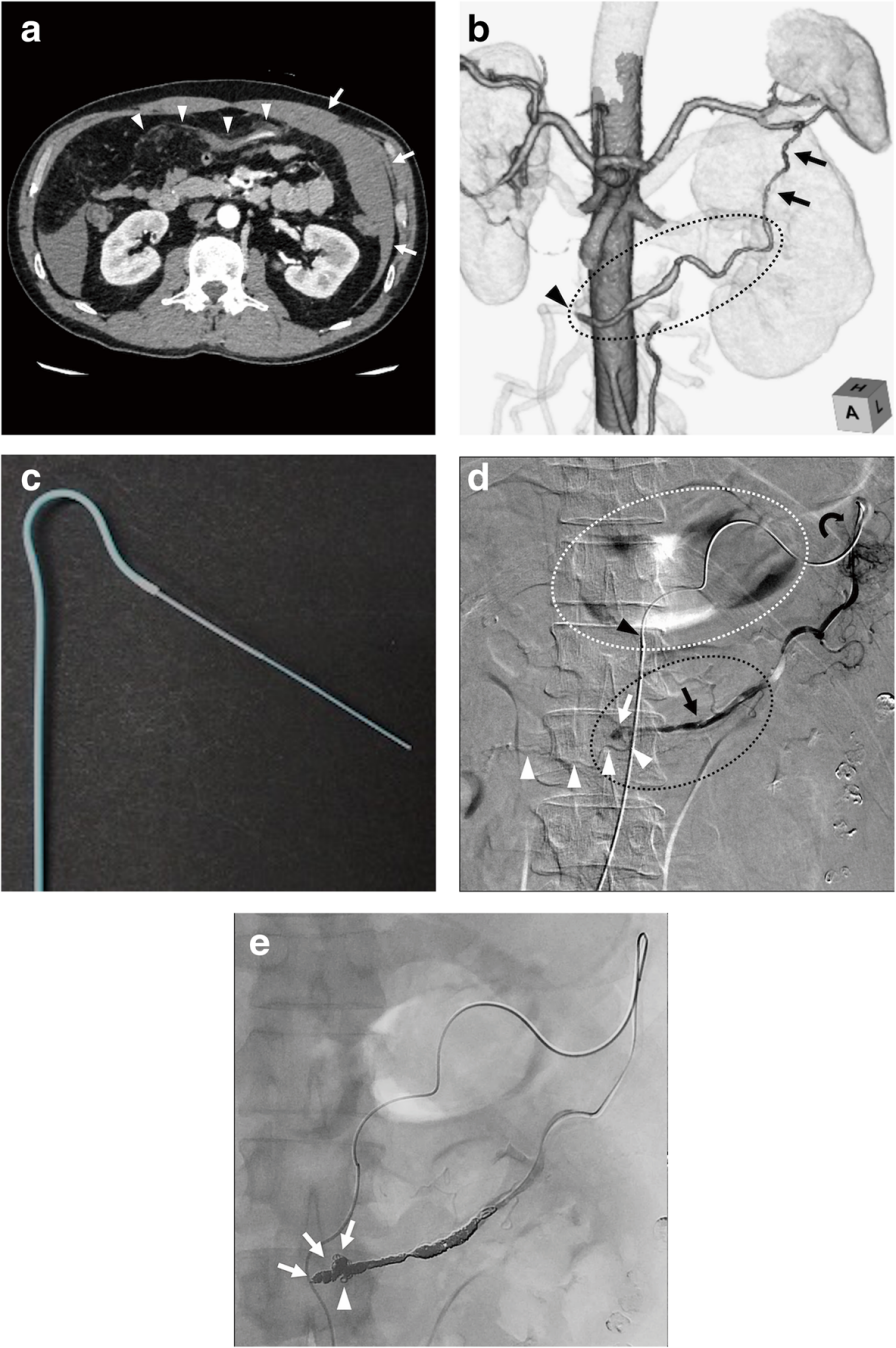
Images from a 55-year-old man with left omental artery (OA) bleeding associated with segmental arterial mediolysis (SAM). **a** Axial contrast-enhanced computed tomography (CT) reveals massive intraperitoneal high-density ascites mainly involving the left portion of the greater omentum (arrows) where the OA with the dilated, stenotic change develops the possible hematoma (arrowheads). **b** Coronal volume-rendered image from CT in the cranial and left anterior oblique projection reveals that the left OA (arrows) arising acutely from a splenic artery branch and coursing away from the greater curvature develops dilated, stenotic change (dotted circle) and that the potential bleeding site at the distal portion of left OA (arrowhead) is far from celiac artery. SAM is the most likely diagnosis, and transarterial embolization is planned. **c** Photograph of the triaxial catheter system consisting of a 4.2 Fr shepherd’s hook catheter, a 2.8 Fr high-flow microcatheter and a 2.0 Fr microcatheter, which serves as the outermost, intermediate, and innermost catheters, respectively. **d** Digital subtraction angiography (DSA) of the left OA in posteroanterior projection using the triaxial catheter reveals a pseudoaneurysm in the distal portion (white arrow). Note an intact distal left. OA runs through (white arrowheads). Coil embolization is planned to isolate the proximal and distal artery (black dotted circle). Note that the SAM-associated left OA courses away from the greater curvature (white dotted circle), which means that this artery differs from left gastroepiploic artery. Note each tip of the triaxial catheter system consisting of the 4.2 Fr shepherd’s hook catheter (black arrowhead), the 2.8 Fr high-flow microcatheter (black curved arrowhead) and the 2.0 Fr microcatheter (black arrow). **e** DSA of the left OA in posteroanterior projection after embolization reveals complete hemostasis. Note that distal omental artery including the pseudoaneurysm (arrows) is densely embolized and that the distal normal left OA is also embolized with metallic coils (arrowhead)

A 55-year-old male with no significant past medical history presented with an acute abdomen. His blood pressure and heart rate were 95/56 mmHg and 55 beats/minute, respectively. CT revealed possible hemorrhagic ascites involving the left portion of the greater omentum and dilated and stenotic change of the left OA with the possible hematoma, which was compatible with a sentinel clot sign (Fig. 1a, b). In addition to such characteristic CT findings, taking into consideration being a middle-aged male without apparent arteriosclerosis, acute-onset symptoms and negative laboratory screening test results and clinical findings suggesting vasculitis, SAM-associated left OA bleeding was suspected, and TAE was planned after written informed consent for the embolization procedure was obtained from the patient.

Celiac artery angiography revealed the dilated and stenotic left OA. Given its acute-angled branching from a splenic artery branch and tortuous and long catheter-trajectory (Fig. 1a, b), we selected a triaxial catheter system consisting of a 4.2 French (Fr) shepherd’s hook catheter, a 2.85 Fr high-flow microcatheter (Carry Leon high-flow microcatheter; UTM, Nagoya, Japan), and a 2.0 Fr microcatheter (Carry Leon microcatheter; UTM, Nagoya, Japan), which served as the outermost, intermediate, and innermost catheters, respectively (Fig. 1c). Left OA angiography revealed the proximal dilated and stenotic change with a distal pseudoaneurysm with an intact distal omental artery branch (Fig. 1d). Taking into consideration the possible collateral circulation from the distal omental artery branch, isolation was successfully performed with 11 pushable coils (10 C-STOPPER Filling COIL, PIOLAX, Yokohama, Japan; 1 Hilal Embolization Microcoil, COOK MEDICAL, Bloomington, IN, USA) and 4 detachable coils (AZUR CX 18, Terumo, Tokyo, Japan) (Fig. 1e). The patient’s hemodynamic status improved, with a blood pressure and heart rate of 122/52 mmHg and 60 beats/minute. During his clinical course in our hospital, he had no abdominal pain or anemia, and he was discharged on hospital day 5. Follow-up CECT after 1 month revealed neither recurrence nor new SAM-associated findings. Based on a follow-up phone call, he has not experienced any symptoms associated with SAM such as abdominal pain during the two-year follow-up.

### Case 2

**Fig. 2:**
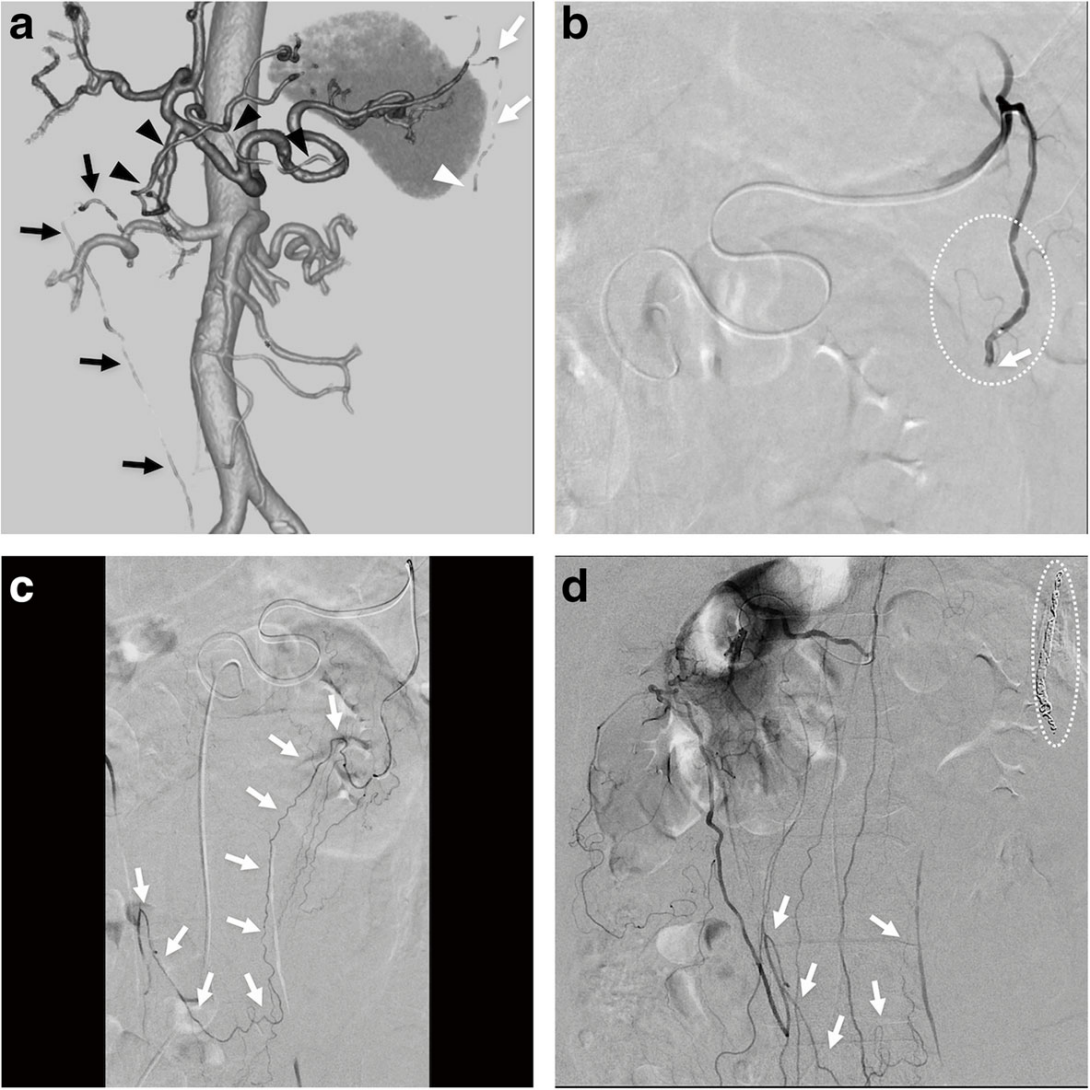
Images from a 60-year-old man with left OA bleeding associated with SAM. **a** Coronal volume-rendered computed tomography image in cranial and right-anterior projection reveals that a left OA (white arrows) arises acutely from a splenic artery branch and develops subtle dilation and stenotic change. The distal side of left OA is completely occluded (white arrowhead), presumably associated with SAM. Note that right gastroepiploic artery (black arrowheads) courses alongside the greater curvature of and one of the right omental branches off vertically (black arrows), which is found to be directly communicated with the to-be embolized left OA (white arrows). SAM is the most likely diagnosis, and transarterial embolization is planned. **b** DSA of left OA in right anterior oblique projection reveals that it acutely arises from a splenic artery and that it develops the dilation and stenotic change (dotted circle) and complete occlusion (arrow). Given that embolization of the proximal left OA at the arrow-indicating point could lead to incomplete hemostasis or rebleeding via arterial collateral network through another OA arising from right gastroepiploic artery, attempt was made to navigate the microcatheter into the distal side beyond the occluded point without complications. **c** DSA of the distal side of left OA in posteroanterior projection reveals that distal left OA does not develop findings suggestive of SAM. Note that the to-be-embolized left OA directly communicates with a right OA arising from right gastroepiploic artery (arrows). Embolization was successfully performed using isolation technique with coils. **d** DSA of gastroduodenal artery in posteroanterior projection reveals a right OA (arrows) directly communicating with the embolized left OA, which does not develop any further a potential bleeding point. Note that the SAM-associated left OA (dotted circle) is embolized with metallic coils. Complete hemostasis by isolation technique with coils is confirmed

A 60-year-old-male with no significant past medical history presented with an acute abdomen. His hemodynamic status was unstable; blood pressure and heart rate were 80/50 mmHg and 111 beats/minute, respectively. CT revealed possible hemorrhagic ascites mainly involving the left greater omentum and dilated, stenotic change of the left OA with the possible hematoma, which was compatible with a sentinel clot sign (Fig. 2a). In addition to such characteristic CT findings, taking into consideration being a middle-aged male without apparent arteriosclerosis, acute-onset symptoms and negative laboratory screening test results and clinical findings suggesting vasculitis, SAM-associated left OA bleeding was suspected, and TAE was planned after written informed consent for the embolization procedure was obtained from the patient.

Celiac artery angiography revealed dilated, stenotic change of the right and left hepatic arteries, right gastroepiploic artery, and left OA. Left OA angiography revealed proximal dilated and stenotic change with distal occlusion (Fig. 2b). Despite having no signs of active bleeding, review of the CT and angiography findings suggested the left OA as the bleeding site because of CT sentinel clot sign. Because left OA was considered as the most likely bleeding site based on CT sentinel clot sign and because he was hemodynamically unstable, TAE was performed. Given that proximal embolization at this point could lead to incomplete hemostasis or rebleeding via arterial collateral network between OAs, an attempt was made to navigate the microcatheter into the distal side beyond the occluded point, which was achieved without complications. The distal OA over the occluded point was intact and directly communicated with a right OA arising from right gastroepiploic artery (Fig. 2c). The SAM-associated lesion was successfully isolated with 13 coils (10 C-STOPPER Filling COILs, PIOLAX, Yokohama, Japan and 3 Tornade Embolisation Coil, COOK MEDICAL, Bloomington, IN, USA). Angiography of the proximal left OA and right gastroepiploic artery confirmed complete hemostasis (Fig. 2d). The patient’s hemodynamic status improved, with a blood pressure and heart rate of 113/75 mmHg and 93 beats/minute. During his clinical course in our hospital, he had no abdominal pain or anemia, and he was transported to another hospital on hospital day 3. Based on a follow-up phone call, he has not experienced any symptoms associated with SAM such as abdominal pain during the one-year follow-up.

## Discussion

The treatment and follow-up strategy can be altered when the differential diagnosis in OA bleeding includes SAM (Heritz et al. 1990; Yasuoka et al. 2008; Rott and Boecker 2018). First, conservative treatment can be selected in SAM-affected OA patients without active bleeding sources. This possible strategy is based on SAM-related vascular findings sometimes resolving spontaneously with conservative treatment (Slavin 2009; Kalva et al. 2011; Ryan et al. 2000; Shimohira et al. 2008). In Case 2 presented herein, conservative treatment without TAE may have resulted in a good clinical course, when considering the negative direct active bleeding finding on CT and angiography. Second, it is important to search for the possible SAM-related vascular findings in the other lesions taking into consideration that SAM can simultaneously and asynchronously involve multiple sites including the vertebral and carotid arteries (Slavin 2009; Kalva et al. 2011; Ryan et al. 2000; Shimohira et al. 2008). In Case 1 presented herein, screening for carotid and vertebral artery involvement using magnetic resonance imaging (MRI) during his hospitalization and follow-up abdominal CT 1 month after the embolization reveal no recurrence or new SAM-associated findings. In Case 2 presented herein, the patient, who was transported to another hospital on hospital day 3, should have undergone follow-up abdominal CT and screening MRI of the carotid and vertebral artery.

SAM-affected OA bleeding can be controlled by TAE with coils. Recently, SAM-affected visceral artery bleeding has been successfully controlled by TAE with coils (Ryan et al. 2000; Shimohira et al. 2008). In the two cases presented here, complete hemostasis was accomplished by TAE with coils. In Case 1, follow-up CECT revealed no complications associated with the embolization such as omental ischemic change. In Case 2, the SAM-associated occluded lesion that was presumably associated with hematoma or dissection could be easily crossed without complication, which led to accomplish isolation with coils. This technique could potentially be applied in the other SAM-affected occluded lesions. Further clinical experience is needed to validate the safety and feasibility of this technique.

**Fig. 3:**
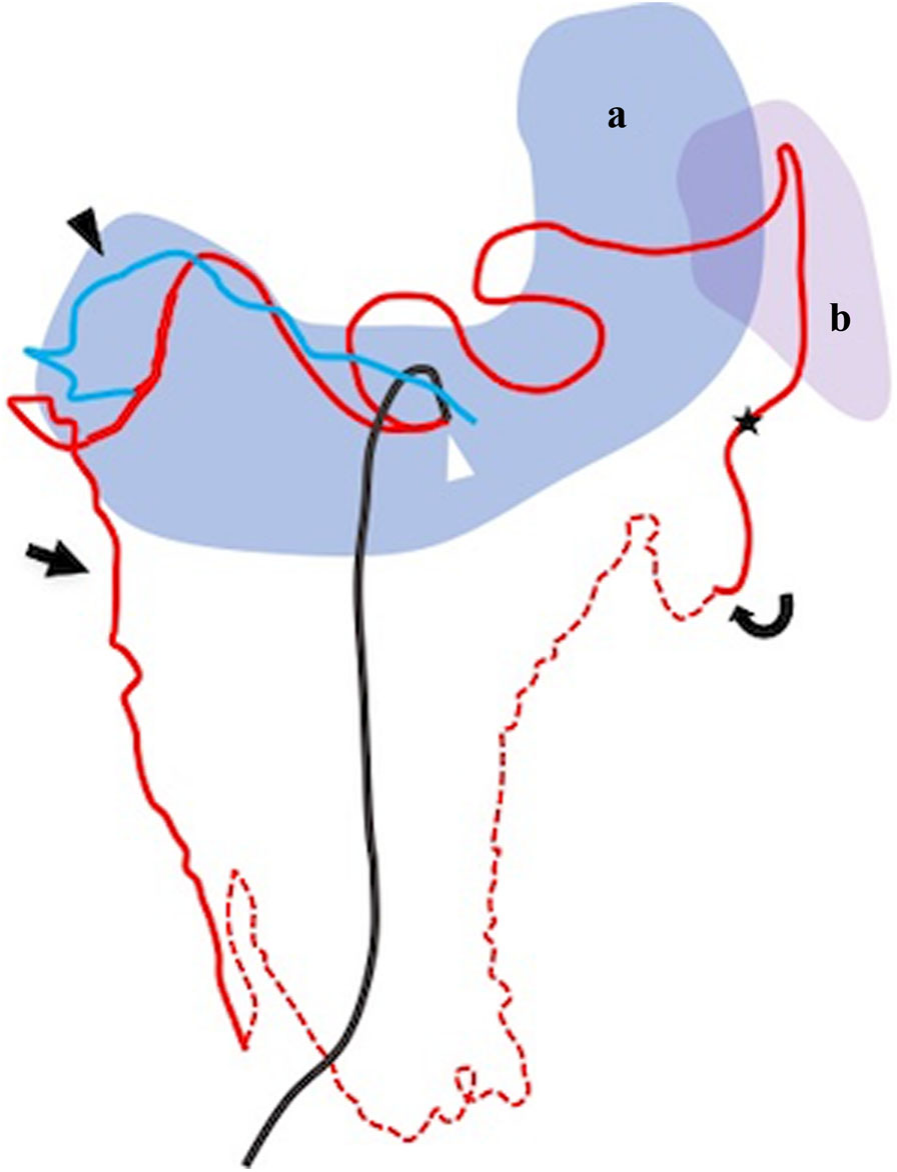
Schematic image of omental arteries and “arcades at the greater omentum” in case 2. The to-be-embolized left omental artery directly communicates with an omental artery branching from the right gastroepiploic artery. Note that proximal embolization at the origin of the left omental artery could cause incomplete hemostasis or rebleeding via collateral circulation from right omental arteries. Black arrow: right omental artery, white arrowhead: the tip of the 4.2 Fr guiding catheter positioned in the celiac artery, black arrowhead: right gastroepiploic artery, black curved arrow: left omental artery, star: the occluded point, red dot line: arcade omental branch between right omental artery and left one, **a**: stomach, **b**: spleen

In previous case reports describing OA bleeding, the culprit bleeding from the OA was not successfully embolized with coils (Rott and Boecker 2018; Tsuchiya et al. 2009; Matsumoto et al. 2011; Takahashi et al. 2012; Tajima et al. 2014; Nishiyama et al. 2018). This failure can be explained by the following two major reasons: the first is failed selective catheterization of the targeted bleeding omental artery; (i) because the omental artery can arise from splenic artery or right gastroepiploic artery with acute angle (Rott and Boecker 2018; Tsuchiya et al. 2009; Matsumoto et al. 2011; Takahashi et al. 2012; Tajima et al. 2014; Nishiyama et al. 2018); (ii) because the omental artery is small in diameter and long-tortuous trajectory (Rott and Boecker 2018; Tsuchiya et al. 2009; Matsumoto et al. 2011; Takahashi et al. 2012; Tajima et al. 2014; Nishiyama et al. 2018); the second is the underrecognized potential for the greater omentum to have arterial collateral network between OAs (Yasuoka et al. 2008; Tsuchiya et al. 2009; Settembre et al. 2018). In Case 1, triaxial catheter system was effective to overcome the difficulties of (i) and (ii) (Kaminou et al. 1998) and then embolization using isolation technique with coils was successfully performed. In Case 2, review of the angiography findings (Fig. 2c and d) confirms that the bleeding left OA directly communicates with another OA arising from right gastroepiploic artery (Fig. 3), which suggests that embolization of proximal left OA could cause rebleeding or incomplete hemostasis via another OA. It is important for radiologists to recognize the potential for the greater omentum to have arterial collateral network between OAs and to try to perform isolation in bleeding OA case.

Although N-butyl cyanoacrylate (NBCA; Histoacryl; B. Braun, Melsungen, Germany) mixed with ethiodized oil (Lipiodol; Guerbet, Aulnay-sous-Bois, France) injection from proximal side can be acceptable in failed selective catheterization of the targeted artery with active bleeding sign (Rott and Boecker 2018; Nishiyama et al. 2018), attention should be paid to NBCA injection-related complications such as the possible proximal embolization, non-target embolization or catheter adhesion associated with its liquid and permanent embolic nature (Takeuchi et al. 2014, Enokizono et al. 2012). Basic embolization procedure with isolation technique with coils should be primarily tried in terms of the safety and feasibility.

## Conclusion

SAM can involve left OA and be controlled by TAE with coils. Trying to cross a SAM-associated occlusion can lead to complete hemostasis. In embolization of left OA, it is important to recognize (i) possible acute-angled branching from splenic artery and the tortuous and long catheter-trajectory and (ii) the potential for the greater omentum to have arterial collateral network between OAs. To overcome these difficulties, using a triaxial catheter system can be effective. Further documentation of clinical experience is needed to validate the safety and feasibility of this technique in future clinical practice.

## Data Availability

The datasets used and/or analyzed during the current study are available from the corresponding author on reasonable request.

## Abbreviations

SAM: Segmental arterial mediolysis
OA: Omental artery
CT: Contrast-enhanced computed tomography
TAE: Transarterial embolization
NBCA: N-butyl cyanoacrylate
